# Functional Decline After Multimodal Treatment of Oral Squamous Cell Carcinoma: A Prospective Longitudinal Study Using the Eastern Cooperative Oncology Group (ECOG) Scale

**DOI:** 10.7759/cureus.117648

**Published:** 2026-10-08

**Authors:** Chaiti Saha, Mahmudul Hasan, Ruman Banik, Ashik Abdullah Imon, Mohidul Islam, Md. Asaduzzaman

**Affiliations:** 1 Oral and Maxillofacial Surgery, Dhaka Dental College and Hospital, Dhaka, BGD; 2 Oral and Maxillofacial Surgery, Bangladesh Medical University, Dhaka, BGD

**Keywords:** adjuvant therapy, ecog performance status, head and neck surgery, oral squamous cell carcinoma, quality of life

## Abstract

Background and aim

Curative treatment of oral squamous cell carcinoma (OSCC) is often physically demanding, and the functional cost of therapy is not always reflected in oncological outcome measures alone. However, prospective evidence describing longitudinal changes in functional performance among patients undergoing curative treatment for OSCC, particularly in South Asian populations, remains limited. This prospective study aimed to evaluate longitudinal changes in Eastern Cooperative Oncology Group performance status among patients undergoing curative surgical treatment for OSCC. Specifically, Eastern Cooperative Oncology Group performance status (ECOG PS) was assessed before treatment, one month after surgery, and one month after completion of adjuvant therapy when indicated. This study also compared changes in performance status across treatment stages and explored clinical factors associated with functional deterioration. We hypothesized that multimodal treatment for OSCC would result in a significant decline in functional performance, with the magnitude of deterioration varying according to baseline performance status, treatment intensity, and reconstructive procedures.

Methods

In this longitudinal study conducted over one year (May 2024 to June 2025) at the Department of Oral and Maxillofacial Surgery, Bangladesh Medical University, we purposively enrolled 36 histopathologically confirmed OSCC patients undergoing curative surgery, with adjuvant therapy as indicated. ECOG PS was assessed by a semi-structured interview before treatment and one month after completion of surgery or, where applicable, one month after adjuvant therapy. We used paired-samples t-tests, Pearson correlations, and chi-square/unpaired Student's t-tests as appropriate (SPSS version 27.0 {Armonk, NY: IBM Corp.}; p<0.05 significant).

Results

Mean age was 55.6±10.7 years; 21 (58.3%) were female, and homemakers formed the largest single occupational group (n=20, 55.5%). A total of 15 patients (41.7%) had surgery alone, 13 (36.1%) had surgery plus radiotherapy, and eight (22.2%) had surgery plus chemoradiotherapy. Mean ECOG PS rose from 0.58±0.60 at baseline to 1.78±1.53 after treatment (mean change: +1.19±1.21; p<0.001). Deterioration occurred in 12 of 15 (80.0%) patients treated with surgery alone, eight of 13 (61.5%) patients receiving surgery plus radiotherapy, and seven of eight (87.5%) patients receiving surgery, radiotherapy, and chemotherapy. Higher baseline PS predicted greater absolute deterioration (R^2^=0.448 between pre- and post-treatment scores). Five patients (13.9%) died; both patients with baseline PS 2 died, compared with three of 34 patients with baseline PS 0-1. Score deterioration was most pronounced after reconstruction with distant free flaps (nine of 10 {90.0%} patients) and least pronounced after local flap reconstruction (three of seven {42.9%} patients).

Conclusion

Curative treatment of OSCC is associated with a clinically meaningful decline in functional performance status, disproportionately affecting patients who already present with impaired baseline status. Pre-treatment ECOG PS should be incorporated into shared decision-making and rehabilitation planning for OSCC patients.

## Introduction

Oral squamous cell carcinoma (OSCC) accounts for approximately 90% of all oral malignancies and remains a major public health challenge worldwide, particularly in South Asia where tobacco smoking, smokeless tobacco, betel quid, and arecanut consumption are highly prevalent [1]. Despite advances in prevention and treatment, the global burden of OSCC continues to increase because of population growth, aging, and persistent exposure to behavioral risk factors [2]. Recent projections estimate that the incidence of oral cancer will increase by nearly 40% by 2040, highlighting the growing demand for effective treatment strategies that not only improve survival but also preserve patients' functional independence [1].

Surgical resection with histologically clear margins remains the cornerstone of curative treatment for resectable OSCC and is frequently combined with cervical lymph node dissection and reconstructive procedures. Depending on pathological risk factors, post-operative radiotherapy or concurrent chemoradiotherapy is recommended to improve locoregional disease control and overall survival [3]. Although these multimodal treatment approaches have substantially improved oncologic outcomes, they often result in significant functional impairment involving speech, mastication, swallowing, shoulder function, and overall physical performance [4]. Consequently, survivorship after OSCC extends beyond disease control and increasingly emphasizes preservation of functional capacity and quality of life.

Functional performance status has become an important patient-centered outcome because it reflects an individual's ability to perform activities of daily living, tolerate cancer treatment, and maintain independence. Among the available assessment tools, the Eastern Cooperative Oncology Group performance status (ECOG PS) scale is one of the most widely used measures in oncology [5]. Originally developed to standardize patient assessment in clinical trials, the ECOG PS provides a simple and reproducible five-point ordinal scale that evaluates physical functioning, self-care ability, and ambulatory status [6]. Beyond its role in treatment selection, ECOG PS has consistently demonstrated prognostic value for treatment tolerance, post-operative recovery, treatment-related toxicity, and overall survival across multiple malignancies [7-9].

In patients with head and neck cancer, impaired performance status has been associated with poorer survival, increased post-operative complications, prolonged hospitalization, and reduced tolerance to adjuvant therapy [10]. Importantly, functional performance is not static but evolves throughout the cancer care continuum. Previous investigations have demonstrated short-term deterioration following major head and neck surgery, partial recovery during subsequent follow-up, and persistent functional deficits in selected patient populations [11]. However, much of the existing evidence has focused on heterogeneous head and neck cancers, retrospective datasets, elderly cohorts, or advanced-stage disease, with relatively limited prospective evaluation specifically examining serial changes in functional performance among patients with OSCC undergoing contemporary multimodal treatment. Furthermore, evidence from South Asian populations, where disease characteristics, treatment patterns, and socioeconomic contexts differ substantially from those reported in high-income countries, remains scarce.

## Materials and methods

Study design and setting

This was a hospital-based, prospective, longitudinal study conducted in the Department of Oral and Maxillofacial Surgery, Bangladesh Medical University (BMU), Dhaka, Bangladesh, over one year (May 2024 to June 2025).

Study population and sampling

All patients with histopathologically confirmed OSCC who were admitted to the department and underwent curative surgical treatment during the study period were eligible. Patients were enrolled by purposive sampling. We used the following formula to calculate sample size [12].

$n = \frac{[Z_&alpha; \sqrt{(P_0 (1-P_0)}+Z_&beta; \sqrt{(P_1 (1-P_1)}]^2} {(P_1-P_0 )^2}$

For the sample size calculation, anticipated proportions of 70% and 90% were used for the proportion of OSCC patients with a given ECOG performance status before and after curative surgical treatment, respectively, based on clinical experience and expert judgment. The minimum sample size was calculated for a two-proportion comparison (Z_α_=1.96, Z_β_=0.84, anticipated proportions P_0_=70%, P_1_=90%). Putting these values into the formula, the estimated sample size was calculated as below.

$n = \frac{[1.96\sqrt{(0.70(1-0.70)}+0.84\sqrt{(0.90(1-0.90)}]^2} {(0.90-0.70)^2} \simeq 34$

To allow for loss to follow-up and incomplete data, the target was increased to 36, which was achieved. Figure 1 summarizes the overall study flow, from patient recruitment to data analysis.

**Figure 1:**
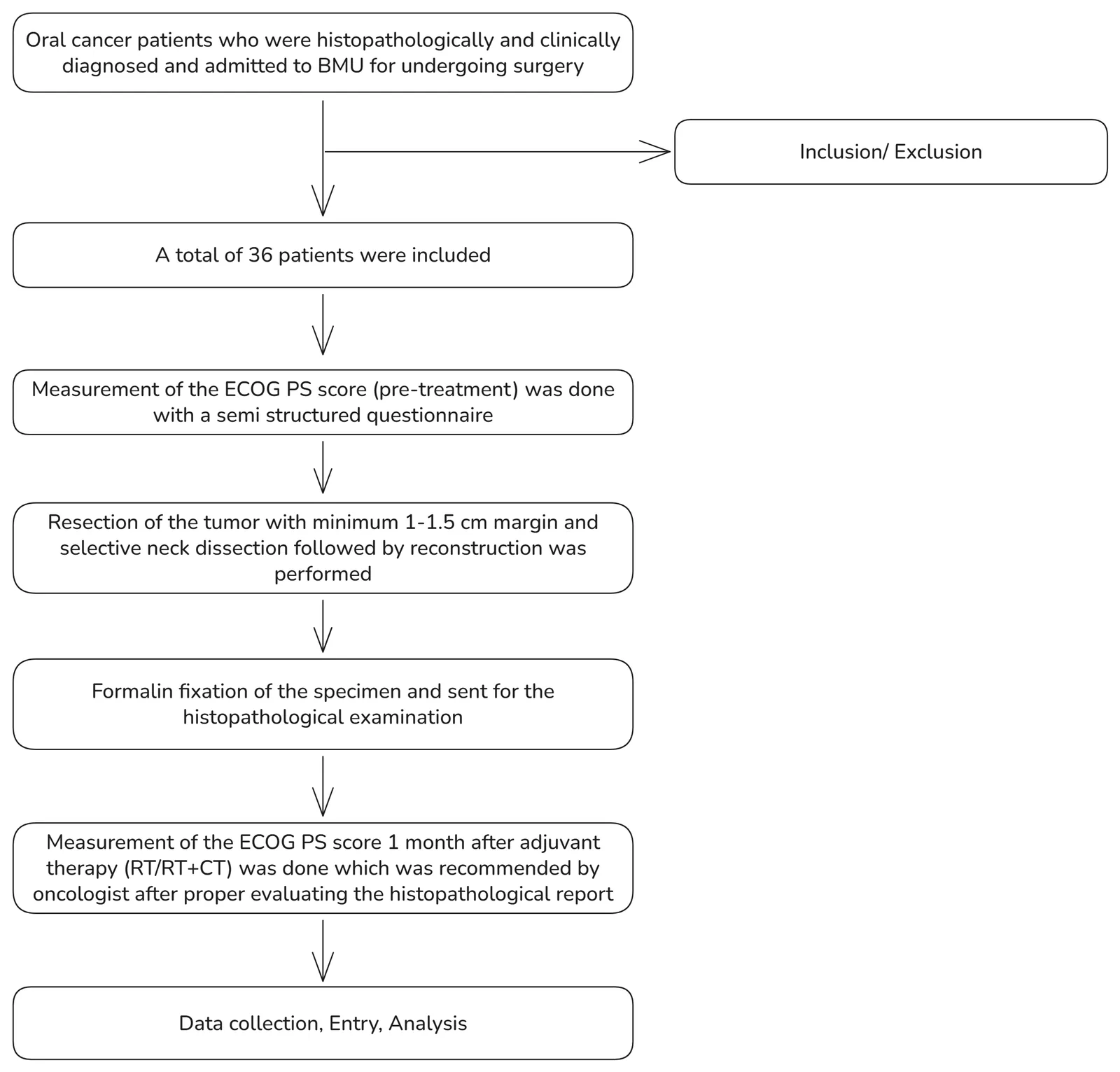
Flow chart showing the study design, patient selection, treatment, and ECOG performance status assessment protocol. ECOG: Eastern Cooperative Oncology Group; ECOG PS: ECOG performance status; BMU: Bangladesh Medical University; RT: radiotherapy

Inclusion and exclusion criteria

Patients were eligible if they were aged ≥18 years, of either sex, had histopathologically confirmed oral squamous cell carcinoma (OSCC), and were scheduled to undergo curative surgical treatment with or without adjuvant therapy. Patients were excluded if they had received prior neoadjuvant therapy and/or chemotherapy prior to surgery, presented with inoperable disease, were unable to complete the planned adjuvant therapy because of social or financial constraints, were unfit for general anesthesia on pre-operative assessment, or were pregnant. Patients with systemic comorbidities, such as diabetes mellitus or hypertension, were eligible for inclusion provided that they were otherwise fit for surgery and met the study eligibility criteria.

Clinical, radiological, and histopathological work-up

Each patient underwent detailed history-taking and clinical examination, followed by incisional biopsy of the suspected lesion for histopathological confirmation and grading. Histopathological assessment and grading were performed by qualified oral and maxillofacial pathologists according to the institution’s standardized diagnostic criteria and reporting protocols. Pre-operative orthopantomography and contrast-enhanced computed tomography and/or magnetic resonance imaging of the maxillofacial region were obtained to assess local extension and nodal status. Clinical tumor and nodal staging followed the American Joint Committee on Cancer (AJCC) Eighth Edition, and this staging guided the surgical plan for primary-site excision, neck dissection, and reconstruction [13].

Surgical and adjuvant treatment

Wide local excision was performed with a minimum 1.0-1.5 cm clinical margin in all dimensions. Selective (supra-omohyoid or extended supra-omohyoid) neck dissection was performed according to nodal status, and reconstruction was selected from the reconstructive ladder, ranging from primary closure and local flaps (e.g., palatal rotation, facial artery musculomucosal, buccal fat pad) to locoregional flaps (pectoralis major myocutaneous, submental island) and distant free flaps (radial forearm, anterolateral thigh, fibular) according to defect size and site. The multidisciplinary team recommended adjuvant radiotherapy or chemoradiotherapy based on post-operative histopathological margin status and nodal involvement, with the treating oncologist determining the specific regimen, dose, and timing.

Assessment of ECOG performance status

ECOG PS was assessed by a semi-structured questionnaire developed in both English and Bangla and administered to participants in Bangla. The assessment covered tolerance of strenuous, moderate, and light activities; independence in daily self-care (feeding, toileting, bathing, dressing, and medication management); ambulation and mobility; and time spent sitting or lying in bed. Patients were scored on the standard 0-5 scale, from 0 (fully active) to 4 (completely disabled); a score of 5 denotes death. Clinical observation was used to complement the interview-based assessment where appropriate. An oral and maxillofacial surgeon conducted all interviews under the research supervisor's supervision throughout the study. ECOG PS was measured at the following three time points: (i) before initiation of treatment; (ii) one month after surgery; and (iii) for patients requiring adjuvant therapy, one month after its completion. For patients receiving adjuvant therapy, the post-treatment score used for the primary before-after comparison was the score recorded one month after completion of the full treatment course (surgery±adjuvant therapy).

Study variables

Demographic variables were age and sex. Independent variables included treatment modality (surgery alone versus surgery with adjuvant therapy), tumor type, clinical stage and grade, nutritional status, comorbidities, and type of reconstructive procedure. The dependent variable was ECOG PS measured one month after completion of treatment.

Statistical analysis

Data were entered and analyzed in SPSS version 27.0 (Armonk, NY: IBM Corp.). Categorical variables were summarized as frequencies and percentages and compared using the chi-square test; continuous variables were summarized as mean±standard deviation and compared using the unpaired Student's t-test. The paired-samples t-test was used to compare ECOG PS before and after treatment within patients. Pearson correlation analysis was used to examine the linear relationship between pre- and post-treatment ECOG PS. A two-tailed p<0.05 was considered statistically significant.

## Results

Baseline characteristics

A total of 36 patients with OSCC were enrolled, of whom 21 (58.3%) were female and 15 (41.7%) were male (Table 1). The mean age was 55.6±10.7 years, with the disease most prevalent in the sixth to seventh decades of life. A total of 15 patients (41.7%) underwent surgery alone, 13 (36.1%) underwent surgery followed by radiotherapy, and eight (22.2%) underwent surgery with chemoradiotherapy; treatment allocation was determined jointly by the operating surgeon and oncologist based on post-operative histopathology and clinico-pathological stage. The occupational profile of the cohort is notable and clinically informative as follows: more than half of the participants (n=20, 55.5%) were homemakers, followed by businessmen (n=7, 19.4%), service holders (n=5, 13.8%), farmers (n=2, 5.6%), and one laborer and one mason (n=1 each, 2.8% each). With respect to treatment, 15 patients (41.7%) were managed with surgery alone, while the majority, 21 patients (58.3%), required adjuvant therapy in addition to surgery - 13 (36.1%) received radiotherapy, and eight (22.2%) received chemoradiotherapy.

**Table 1:** Characteristics of the study cohort (n=36).

Characteristics	Frequency (n)	%
Sex		
Female	21	58.3
Male	15	41.7
Age (years)		
31-50	15	41.7
51-70	19	52.8
>70	2	5.6
Mean±SD	55.6±10.7	-
Occupation		
Businessman	7	19.4
Homemaker	20	55.5
Service	5	13.8
Farmer	2	5.6
Labor	1	2.8
Mason	1	2.8
Treatment received		
Surgery alone	15	41.7
Surgery+radiotherapy	13	36.1
Surgery+chemoradiotherapy	8	22.2

Change in ECOG performance status

At baseline, 17 patients (47.2%) had ECOG PS 0, 17 (47.2%) had PS 1, and two (5.6%) had PS 2; no patient presented with PS 3 or 4. Mean baseline ECOG PS was 0.58±0.60. One month after completion of treatment, only five patients (13.9%) remained at PS 0, 15 (41.7%) were at PS 1, 10 (27.8%) were at PS 2, one (2.8%) was at PS 4, and five (13.9%) had died (recorded as PS 5). Mean post-treatment ECOG PS was 1.78±1.53, a mean increase of 1.19±1.21 points compared with baseline (paired t-test, p<0.001), indicating statistically significant deterioration in functional status attributable to the aggregate burden of curative treatment (Table 2).

**Table 2:** ECOG performance status before and one month after completion of treatment (n=36). *Paired-samples t-test. ECOG: Eastern Cooperative Oncology Group; ECOG PS: ECOG performance status

ECOG PS score	ECOG PS	Baseline, n (%)	Post-treatment, n (%)	p-Value
0	Fully active	17 (47.2)	5 (13.9)	p<0.001*
1	Restricted strenuous activity	17 (47.2)	15 (41.7)	
2	Ambulatory, self-care only	2 (5.6)	10 (27.8)	
3	Limited self-care	0 (0)	0 (0)	
4	Completely disabled	0 (0)	1 (2.8)	
5	Death	0 (0)	5 (13.9)	
Mean±SD		0.58±0.60	1.78±1.53	

Performance status change by treatment modality

The magnitude and pattern of functional deterioration varied by treatment modality (Table 3). Among patients treated with surgery alone (n=15), 12 (80.0%) showed deterioration, most commonly by one point (nine, 60.0%), with a smaller proportion worsening by two or three points (one, 6.7% and two, 13.3%, respectively). Among patients receiving surgery plus radiotherapy (n=13), eight (61.5%) deteriorated, predominantly by one point (six, 46.2%), with isolated cases worsening by two or four points. Among patients receiving surgery, radiotherapy, and chemotherapy (n=8), the most intensive treatment group, seven (87.5%) deteriorated, the highest proportion of any subgroup, including two patients (25.0%) who worsened by four points, the largest single-group shift observed in the study.

**Table 3:** Change in ECOG performance status by treatment modality (n=36). ECOG: Eastern Cooperative Oncology Group

Treatment modality	No change, n (%)	Any deterioration, n (%)	1 point, n (%)	2 points, n (%)	3 points, n (%)	4 points, n (%)
Surgery alone (n=15)	3 (20)	12 (80)	9 (60)	1 (6.7)	2 (13.3)	0 (0)
Surgery+radiotherapy (n=13)	5 (38.5)	8 (61.5)	6 (46.2)	1 (7.7)	0 (0)	1 (7.7)
Surgery+radiotherapy+chemotherapy (n=8)	1 (12.5)	7 (87.5)	4 (50.0)	0 (0)	1 (12.5)	2 (25.0)

Influence of baseline performance status on deterioration

The magnitude of deterioration was closely related to baseline status. Among the 17 patients who began at PS 0, five (29.4%) showed no change, nine (52.9%) worsened by one point, and three (17.6%) worsened by two points (mean change: +0.88±0.69). Among the 17 patients who began at PS 1, six (35.3%) showed no change, seven (41.2%) worsened by one point, three (17.6%) worsened by two points, and one (5.9%) worsened by three points (mean change: +1.29±1.49). Both patients who began at PS 2 deteriorated by three points, reaching PS 5 (death) in both cases. A scatter plot of pre-operative versus one-month post-treatment ECOG PS showed a positive linear relationship (coefficient of determination {R^2^}=0.448), confirming that a higher baseline score was associated with proportionally greater absolute deterioration after treatment (Figure 2).

**Figure 2:**
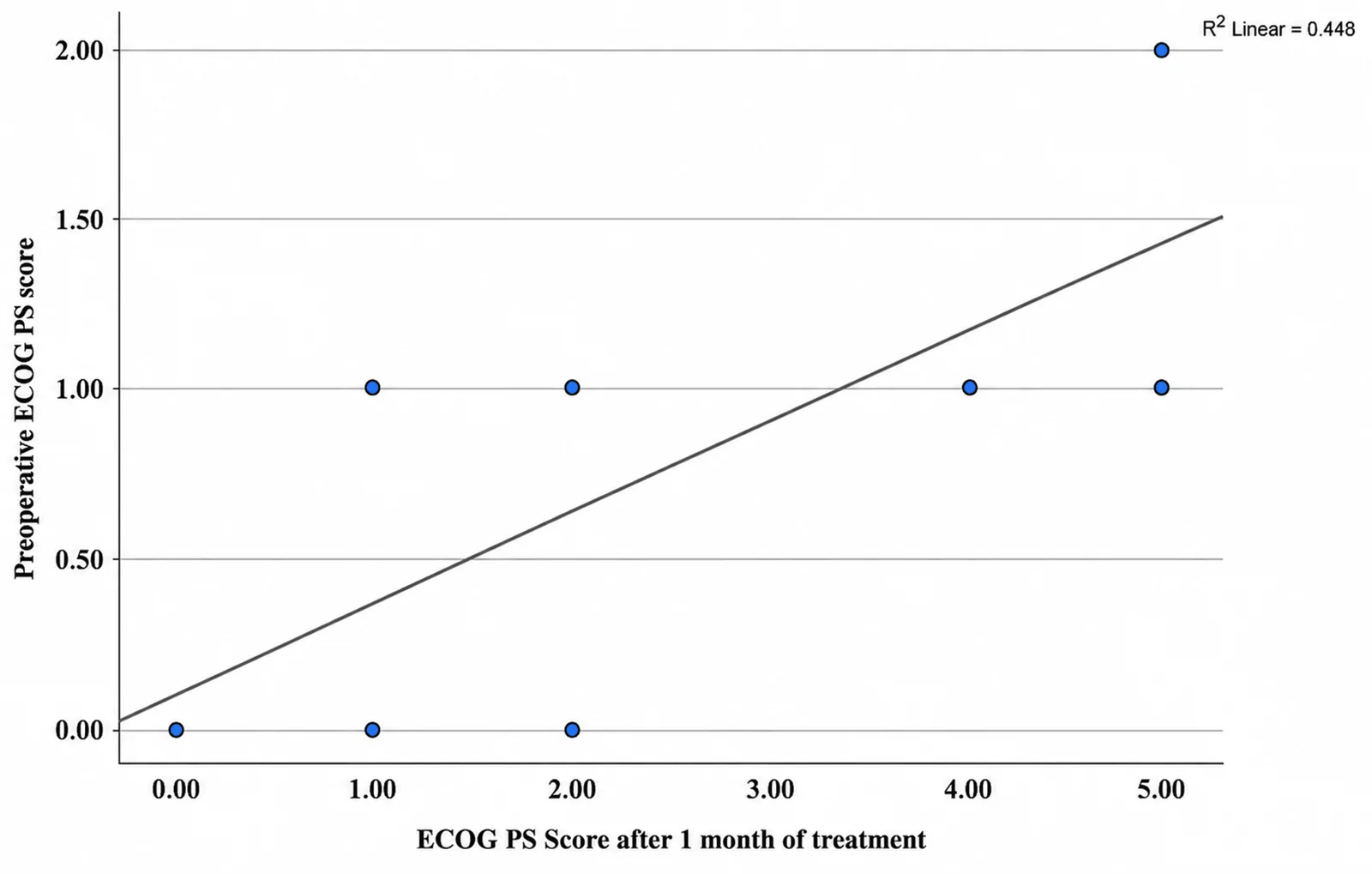
Relationship between pre-operative ECOG performance status and ECOG performance status one month after completion of treatment (n=36; R²=0.448). ECOG: Eastern Cooperative Oncology Group; ECOG PS: ECOG performance status

Mortality

Five patients (13.9%) died during the observation period. Three (60%) had a baseline PS of 1 and two (40%) had a baseline PS of 2; thus, both patients (100%) with PS 2 died, compared with three of the remaining 34 patients (8.8%) with PS 0 or 1. Patients with poor pre-treatment performance status who died developed post-operative pulmonary complications, whereas patients with good baseline performance status who died remained functionally stable after surgery but subsequently died from complications of adjuvant chemoradiotherapy.

Performance status change by reconstruction type

Deterioration was most frequent among patients reconstructed with a distant free flap (radial forearm, anterolateral thigh, or fibular free flap; n=10), nine (90%) of whom showed score deterioration (five {50%} by one point; one {10%} by two points; two {20%} by three points; and one {10%} by four points) (Table 4). Among 11 patients managed with primary closure, nine (81.8%) deteriorated, uniformly by one point. Among seven patients reconstructed with a local flap, four (57.1%) showed no change, and three (42.9%) deteriorated by one point. Among eight patients reconstructed with a locoregional flap (pectoralis major myocutaneous or submental island flap), six (75%) deteriorated (three {37.5%} by one point; one {12.5%} by three points; and two {25%} by four points).

**Table 4:** ECOG performance status deterioration by method of surgical reconstruction (n=36). ECOG: Eastern Cooperative Oncology Group

Reconstruction type	n	Patients with any PS deterioration, n (%)
Primary closure	11	9 (81.8)
Local flap	7	3 (42.9)
Locoregional flap	8	6 (75)
Distant free flap	10	9 (90)

## Discussion

In this cohort of 36 patients treated for oral squamous cell carcinoma (OSCC), mean ECOG performance status rose significantly from 0.58±0.60 before treatment to 1.78±1.53 one month after treatment completion (p<0.001), a mean deterioration of 1.19±1.21 points. Perhaps the most clinically telling single statistic in this dataset is that 47.2% of patients presented fully active (ECOG PS 0), yet only 13.9% retained that status after treatment. In other words, curative-intent therapy for OSCC did not simply push already vulnerable patients further into disability; it measurably eroded the functional reserve of patients who were, by conventional criteria, considered fit for surgery. This finding reframes "successful" oncological treatment as one that is rarely functionally cost-free, even in a well-selected surgical population [14].

The demographic profile of this cohort adds useful context to that finding. Mean age was 55.6±10.7 years, and more than half of participants (52.8%) fell within the 51-70-year band, that is, predominantly in the sixth and seventh decades of life, somewhat older than the fifth-decade peak reported in several other South Asian series [15].

The female predominance observed in this cohort (58.3%) differs from the male predominance typically reported in South Asian OSCC series, most of which are driven by higher rates of smoked and smokeless tobacco use among men [16]. The occupational profile of this cohort offers a plausible, data-grounded explanation for this discrepancy rather than leaving it as an unexplained anomaly; 55.5% of participants were homemakers, a group that in this population was almost exclusively female. This raises the possibility that domestic or home-based exposure to smokeless tobacco and arecanut (betel quid) use, well-documented but often under-recognized risk factors among South Asian women, contributed disproportionately to disease burden in this cohort [17]. This remains an inference rather than a demonstrated causal pathway, since habit-specific exposure data were not systematically collected, but it is a more defensible hypothesis than referral bias alone, and it merits dedicated habit-history data collection in future work.

The relationship between treatment intensity and functional deterioration in this cohort was informative but not straightforwardly linear. Patients treated with surgery alone showed deterioration in 80.0% of cases, those receiving surgery plus radiotherapy showed deterioration in 61.5% of cases, and those receiving the most intensive regimen, surgery, radiotherapy, and chemotherapy, showed the highest rate of deterioration at 87.5%. The finding that triple-modality treatment produced the greatest functional cost is consistent with a cumulative-toxicity model, in which the systemic effects of chemotherapy compound the mucosal and fibrotic effects of radiotherapy on top of the anatomical deficit created by surgery [18]. However, the intermediate position of the surgery plus radiotherapy group, showing less deterioration than surgery alone, is counterintuitive and should not be overinterpreted. With only 13 and 15 patients respectively in these two groups, the difference may reflect case mix rather than a genuine protective effect of radiotherapy as follows: patients selected for surgery alone may, for instance, have included a higher proportion of extensive or anatomically unfavorable resections that were judged not to need adjuvant therapy specifically because of their functional risk, while those selected for combined surgery-radiotherapy may have had more favorable primary defects. This pattern reminds us that treatment modality reflects underlying disease and patient selection as much as an independent exposure, and should be interpreted cautiously given the small subgroup sizes.

The relationship between baseline performance status and the magnitude of deterioration was more consistent and, in our view, the more clinically actionable finding of this study. Patients who began treatment at ECOG PS 0 declined by a mean of 0.88±0.69 points, those beginning at PS 1 declined by 1.29±1.49 points, and both patients who began at PS 2 declined by the maximum possible three points, reaching PS 5 (death). The positive linear relationship between pre- and post-treatment scores (R^2^=0.448) confirms that patients with even mild baseline impairment had disproportionately less physiological reserve to absorb the combined insult of surgery, neck dissection, reconstruction, and adjuvant therapy. This pattern is consistent with a substantial body of oncology literature establishing baseline performance status as a marker of reduced physiological reserve and an independent predictor of adverse outcomes across tumor types [19]. In a meta-analysis of patients with advanced non-small cell lung cancer treated with immune checkpoint inhibitors, a baseline ECOG PS of 2 or worse was associated with substantially worse overall survival (pooled hazard ratio: 2.72, 95% CI: 2.03-3.63) [20]. Similarly, in a large cohort of patients with head and neck squamous cell carcinoma, poor ECOG PS at presentation was independently associated with reduced overall survival alongside tumor size and nodal metastasis [10].

That both of our patients presenting with baseline PS 2 ultimately died is consistent with the broader literature and, in absolute terms, considerably more severe. This underscores that impaired baseline performance status identifies a small but extremely high-risk subgroup for whom standard-intensity curative treatment may cause more functional and survival harm than benefit [21-23]. Whether treatment intensity should be deliberately modified for such patients, accepting a possible trade-off in oncological control for a reduction in treatment-related mortality and functional decline, is a question this study cannot answer directly, given that only two such patients were included. However, larger cohorts with adequately represented PS ≥2 subgroups are well positioned to address this question.

It is worth noting that functional decline after major surgical intervention is not inevitable or necessarily permanent in all clinical contexts. In a very different setting, liver transplantation, Karnofsky performance status has been shown to fall around the time of major surgery but then improve significantly during recovery, with the trajectory of that improvement itself carrying independent prognostic value [9]. This raises the possibility that part of the deterioration captured in our one-month assessment reflects an acute post-operative recovery phase rather than a fixed, permanent functional ceiling. Unlike liver transplantation, however, OSCC surgery does not correct an underlying physiological derangement; it creates a new anatomical and functional deficit (in swallowing, speech, and mastication) as an intended consequence of oncological clearance, and any adjuvant therapy that follows adds further, largely non-reversible mucosal and systemic toxicity.

The association between reconstruction type and degree of functional deterioration followed a biologically plausible gradient, with the highest rates of decline among patients reconstructed with distant free flaps (90.0%) and primary closure (81.8%) and the lowest among those reconstructed with local flaps (42.9%) [24]. Free-flap reconstruction is generally reserved for the largest composite defects, is associated with the longest operative time and additional donor-site morbidity, and is often combined with more extensive neck dissection, each of which independently increases the physiological burden of treatment [25]. The comparatively high deterioration rate after primary closure is less immediately intuitive, since these defects are by definition smaller; however, if the defect involves a functionally critical structure (for example, tongue mobility, or oral competence), even a small resection can produce a disproportionate functional deficit despite requiring only simple closure. As with treatment modality, reconstruction type in this analysis largely serves as a surrogate for defect size, location, and disease extent rather than an independent causal exposure, and the small number of patients in each reconstruction category (7-11 per group) limits the precision of these estimates.

Adjuvant therapy appears to have been an important and largely independent driver of functional decline and mortality in this cohort. Notably, patients who died despite remaining functionally stable through surgery died as a result of complications related to adjuvant chemoradiotherapy, not surgery itself, whereas patients with poor baseline performance status died from post-operative pulmonary complications. This distinction suggests the following two largely separate mechanisms of harm operating in this population: peri-operative/respiratory risk concentrated in patients with pre-existing functional limitation, and systemic treatment toxicity affecting even patients who tolerated surgery well; it reinforces that the functional cost of OSCC treatment cannot be attributed to the surgical component alone. This is consistent with the recognized toxicity profiles of both chemotherapy and radiotherapy in head and neck cancer more broadly [26,27].

Baseline ECOG performance status should be routinely assessed to guide treatment planning, pre-operative counseling, and supportive care in patients with OSCC. Patients with poorer baseline performance status may benefit from multidisciplinary evaluation [13,28]. Future multicenter prospective studies with larger sample sizes, longer follow-up, and validated assessment tools are needed to confirm these findings and evaluate long-term functional recovery.

Several limitations temper the interpretation of these findings. First, this study used purposive sampling, which may introduce selection bias and limits generalizability to other populations and healthcare settings. Second, the overall sample size, and in particular the very small number of patients with baseline ECOG PS 2 (n=2) and the complete absence of patients with baseline PS 3-4, meant that subgroup comparisons, including the treatment-modality and reconstruction-type analyses presented here, were based on small cell counts and should be regarded as descriptive and hypothesis-generating rather than definitive. The non-monotonic pattern observed across treatment-modality subgroups illustrates the risk of over-interpreting proportions derived from groups of seven to 15 patients. Third, although all patients underwent surgery at the same institution, adjuvant therapy was frequently delivered at external centers with potentially variable quality, dosing adherence, and supportive-care standards, which may have introduced unmeasured heterogeneity into post-treatment outcomes. Fourth, several variables that plausibly influence functional trajectory, tumor stage and grade, sub-site, nutritional status, and comorbidity burden, were collected as part of the study design but were not analyzed in relation to performance-status change; incorporating these variables in future analyses of this or similar datasets would substantially strengthen causal inference regarding the drivers of functional decline. Fifth, the ECOG PS assessment tool used, while based on the standard scale, was administered via a semi-structured interview that has not been formally validated for this population, which may affect measurement reliability and limit comparability with studies using validated instruments. Finally, follow-up was limited to one month after treatment completion; as discussed above, this window cannot distinguish an acute, potentially reversible post-operative recovery phase from a fixed, permanent functional deficit, and longer prospective follow-up is needed to characterize the true long-term functional trajectory of OSCC survivors.

## Conclusions

Curative treatment of oral squamous cell carcinoma, surgery with or without adjuvant radiotherapy or chemoradiotherapy, is associated with a significant decline in functional performance status within the first month after treatment. This decline is not confined to patients with poor baseline status; even well-selected surgical candidates who begin treatment fully active bear a measurable functional cost. The magnitude of deterioration is closely tied to baseline performance status, treatment intensity, and reconstruction burden, with the poorest baseline status associated with the worst outcomes. These findings support routine, structured assessment of ECOG performance status before treatment planning, both to guide realistic patient counseling and to identify those who may benefit from closer peri-operative surveillance, adjuvant-phase monitoring, or reconsideration of treatment intensity.
